# Associação entre os Níveis Séricos se Serglicina e o Infarto do Miocárdio com Supradesnivelamento do Segmento ST

**DOI:** 10.36660/abc.20190554

**Published:** 2021-04-08

**Authors:** Burcu Ugurlu Ilgın, Emrullah Kızıltunç, Murat Gök, Ender Ornek, Canan Topcuoglu, Mustafa Çetin, Orhan Karayiğit

**Affiliations:** 1 TC Saglık Bakanlıgı Gazi Mustafa Kemal Devlet Hastanesi Ankara Turquia TC Saglık Bakanlıgı Gazi Mustafa Kemal Devlet Hastanesi – Cardiology, Ankara - Turquia; 2 Edirne Provincial Health Directorate Edirne Sultan 1st Murat State Hospital Cardiology Department Edirne Turquia Cardiology Department, Edirne Provincial Health Directorate Edirne Sultan 1st Murat State Hospital, Edirne - Turquia; 3 Numune Education and Research Hospital Medical Biochemistry Department Ankara Turquia Medical Biochemistry Department, Numune Education and Research Hospital, Ankara - Turquia; 4 Numune Education and Research Hospital Cardiology Department Ankara Turquia Cardiology Department, Numune Education and Research Hospital, Ankara –Turquia

**Keywords:** Doenças Cardiovasculares, Infarto do Miocárdio, Aterosclerose, Doença Arterial Coronariana, Inflamação, Biomarcadores, Serglicina

## Abstract

**Fundamento:**

Sugere-se que a serglicina tenha funções importantes na estabilização da fibrina e inflamação, mas há informações limitadas sobre seu valor clínico para a doença cardíaca aterosclerótica.

**Objetivo:**

O objetivo do presente estudo é descobrir os níveis séricos de serglicina em pacientes com infarto agudo do miocárdio e nos indivíduos do grupo controle; e investigar a associação entre os níveis de serglicina com marcadores de inflamação e marcadores de tamanho do infarto.

**Métodos:**

A população do estudo consistiu em 75 pacientes com infarto do miocárdio com supradesnivelamento do segmento ST (IAMCSST) e 57 pacientes com artérias coronárias normais (NCA) (grupo controle). As características dos pacientes, os níveis séricos de serglicina, os níveis de proteína C-reativa ultrassensível (PCR-us), os níveis máximos de troponina T e outros parâmetros bioquímicos foram registrados. O valor de p<0,05 foi considerado estatisticamente significativo.

**Resultados:**

O grupo controle consistiu em indivíduos mais jovens e que fumam menos do que os do grupo IAMCSST. O número de mulheres no grupo controle foi maior do que no grupo IAMCSST. Os níveis séricos de serglicina foram significativamente maiores no grupo IAMCSST do que no grupo controle (102,81±39,42 vs. 57,13±32,25, p<0,001). As análises de correlação revelaram uma correlação positiva significativa entre a serglicina e a troponina (correlação de postos de Spearman: 0,419; p<0,001) e entre a serglicina e a proteína C-reativa ultrassensível (correlação de postos de Spearman: 0,336; p<0,001). A análise de regressão logística multivariada demonstrou que os níveis séricos de serglicina apresentaram-se independentemente associados com IAMCSST. Usando um nível de corte de 80,47 *μ*g/L, o nível de serglicina foi preditor da presença de IAMCSST com uma sensibilidade de 75,7% e especificidade de 68,4%.

**Conclusão:**

Os níveis séricos de serglicina apresentaram-se significativamente maiores no grupo IAMCSST do que no grupo controle. Os níveis de serglicina sérica mostraram-se positivamente correlacionados com os níveis de proteína C-reativa ultrassensível e troponina.

## Introdução

A doença cardíaca aterosclerótica é uma das causas mais importantes de morte e morbidade em todo o mundo. A inflamação vascular crônica é aceita na formação da placa aterosclerótica, mas os promotores e impulsionadores da inflamação vascular crônica ainda estão sob investigação.^1^^,^^2^

A serglicina é um proteoglicano intracelular expresso principalmente em neutrófilos, linfócitos, monócitos, macrófagos, plaquetas, megacariócitos e mastócitos,^3^ mas também pode ser produzida por certas células não hematopoiéticas, como as células endoteliais.^4^ É armazenada em vesículas celulares e reage com mediadores como citocinas, quimiocinas, fatores de crescimento e proteases^3^. Existem evidências sobre o papel da serglicina na inflamação e nas cascatas aterogênicas-pró-trombóticas. Demonstrou-se que a síntese e secreção da serglicina são desencadeadas em células endoteliais e monócitos humanos por estimulantes pró-inflamatórios.^5^^,^^6^ Em outro estudo, verificou-se que a serglicina se liga aos receptores C1q e afeta a polimerização da fibrina na formação do coágulo de fibrina.^7^ A serglicina é um dos ingredientes dos grânulos de plaquetas alfa. Esses grânulos estão envolvidos na ativação plaquetária em resposta à inflamação, formação de trombos e aterosclerose.^8^

Os referidos efeitos e funções da serglicina levam a grandes suspeitas sobre a possível relação entre a serglicina e a doença cardiovascular aterosclerótica, mas não há boas evidências. Portanto, este estudo teve como objetivo investigar os níveis séricos de serglicina em pacientes com infarto do miocárdio com supradesnivelamento do segmento ST (IAMCSST) e avaliar a associação entre os níveis séricos de serglicina e marcadores prognósticos de IAMCSST.

## Métodos

### População do estudo

Incluímos pacientes com infarto agudo do miocárdio com supradesnivelamento do segmento ST (IAMCSST) e pacientes com artérias coronárias normais neste estudo transversal unicêntrico entre novembro de 2017 e março de 2018 no Numune Education and Research Hospital, Ancara, Turquia. O protocolo do estudo foi aprovado pelo comitê de ética local e formulários de consentimento informado foram obtidos de todos os participantes.

O diagnóstico de IAMCSST foi feito de acordo com a terceira definição universal do documento sobre infarto do miocárdio.^9^ Todos os pacientes com IAMCSST foram submetidos a intervenção coronária percutânea primária e receberam tratamento médico orientado de acordo com os conhecimentos científicos contemporâneos. Os pacientes submetidos à angiografia coronária eletiva e que apresentavam artérias coronárias normais foram incluídos no estudo como grupo controle. Todos os pacientes com IAMCSST e pacientes com artérias coronárias normais foram recrutados consecutivamente no estudo. Pacientes com síndrome coronariana aguda sem diagnóstico de IAMCSST foram excluídos do estudo. Pacientes com algum distúrbio hematológico, doença inflamatória crônica, acidente vascular cerebral prévio, doença arterial coronariana estável, insuficiência cardíaca, doença renal, doença hepática, malignidade, doença reumatológica, infarto do miocárdio prévio ou histórico de cirurgia arterial coronariana também foram excluídos. Realizou-se ecocardiografia transtorácica em todos os pacientes. Calculou-se a fração de ejeção ventricular esquerda pelo método de Simpson.

### Exames laboratoriais

Todas as amostras de sangue para análise de serglicina foram coletadas dos pacientes após a angiografia, em tubos simples, e o soro foi separado por centrifugação a 4000 rpm por 10 min e armazenado a −80 °C. Contagens sanguíneas diferenciais completas foram determinadas em amostras de sangue venoso periférico obtidas no momento da internação. Utilizou-se um analisador automático para medir os níveis de troponina, proteína C-reativa ultrassensível (PCR-us), colesterol total, triglicerídeos, creatinina e colesterol de lipoproteína de baixa e alta densidade. Os níveis de serglicina sérica foram medidos por um kit de ensaio de imunoabsorção enzimática para serglicina humana (lote no.: E17-109S01, BioVendor Research and Diagnostic Products, 62100 Bmo, República Tcheca). Todas as amostras foram processadas simultaneamente.^5^ Os coeficientes de variação (CV) do kit foram 3,7% e 2,9% para as concentrações de 57,77 ng/mL e 81,57 ng/mL, respectivamente, e a sensibilidade foi de 9,5 ng/mL.

### Análise estatística

Utilizou-se o software SPSS 22.0 para realizar todas as análises estatísticas. A distribuição das variáveis foi analisada pelo teste de Kolmogorov-Smirnov. Os dados contínuos foram apresentados como média ± desvio padrão ou mediana com intervalos interquartílicos, dependendo do padrão de distribuição. O teste t de amostras independentes foi utilizado para comparar variáveis contínuas paramétricas e o teste U de Mann-Whitney foi utilizado para comparar variáveis contínuas não paramétricas. As variáveis categóricas foram comparadas pelo teste do qui-quadrado e expressas em porcentagem. A correlação entre a proteína C-reativa ultrassensível e os níveis de serglicina foi avaliada pelo teste de Spearman. Para a análise multivariada, os possíveis fatores identificados na análise univariada foram posteriormente inseridos na análise de regressão logística para determinar os preditores independentes de infarto do miocárdio. A capacidade dos níveis séricos de serglicina em predizer o IAMCSST foi analisada pela curva ROC (*receiver operating characteristic*). Ao avaliar a área sob a curva, utilizou-se nível de erro tipo I de 5% para aceitar um valor preditivo estatisticamente significativo da variável de teste. Como não havia dados sobre os níveis de serglicina em pacientes com doença arterial coronariana na literatura em inglês, não foi possível calcular o tamanho da amostra antes do estudo.

## Resultados

**Figura 1:**
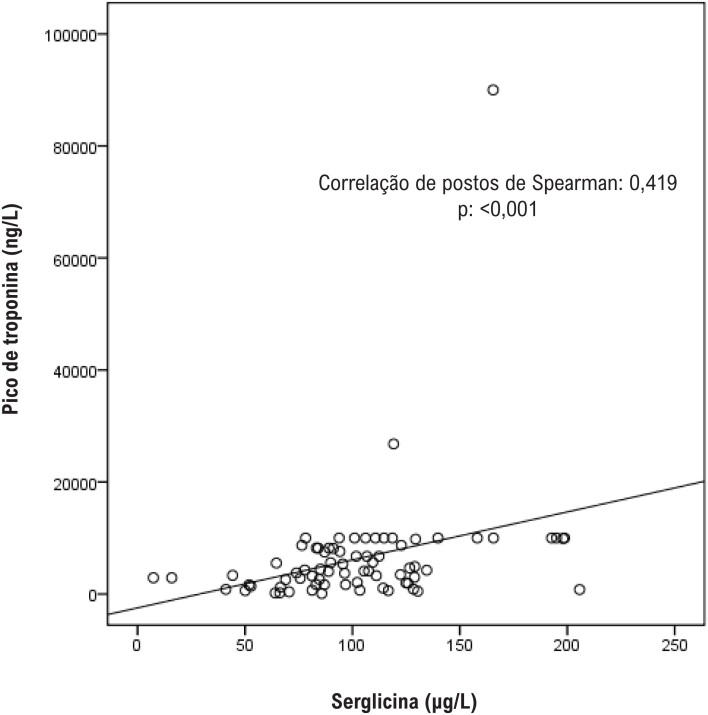
Correlações entre o nível de serglicina plasmática e o nível de troponina em pacientes com IAMCSST. Houve uma correlação significativamente positiva entre o nível de serglicina plasmática e o nível de troponina em pacientes com IAMCSST (r=0,419, p<0,001).

Um total de 132 pacientes (75 IAMCSST e 57 ACN) foram incluídos no estudo. As características clínicas e os parâmetros bioquímicos dos grupos IAMCSST e controle são apresentados na Tabela 1. A proporção de pacientes do sexo masculino e a taxa de tabagismo foram maiores no grupo IAMCSST. Os pacientes eram mais jovens no grupo controle do que no grupo IAMCSST. Os níveis séricos de serglicina mostraram-se significativamente maiores no grupo IAMCSST do que no grupo controle (Tabela 1). Os níveis de serglicina sérica mostraram-se significativamente correlacionados com os níveis de troponina (r=0,419, p<0,001) e proteína C-reativa ultrassensível (r=0,336, p<0,001; Figura 1 e 2). As análises de regressão logística revelaram que o sexo (masculino), o nível de glicemia em jejum, os níveis de proteína C-reativa ultrassensível e serglicina foram preditores independentes de IAMCSST (Tabela 2). Realizou-se a análise ROC para determinar a capacidade do nível de serglicina para predizer IAMCSST. A área sob a curva foi de 0,809 (intervalo de confiança de 95%: 0,737–0,881; p<0,001). Usando um nível de corte de 80,47 μg/L, o nível de serglicina previu a presença de IAMCSST com uma sensibilidade de 75,7% e especificidade de 68,4% (Figura 3).

**Figura 2:**
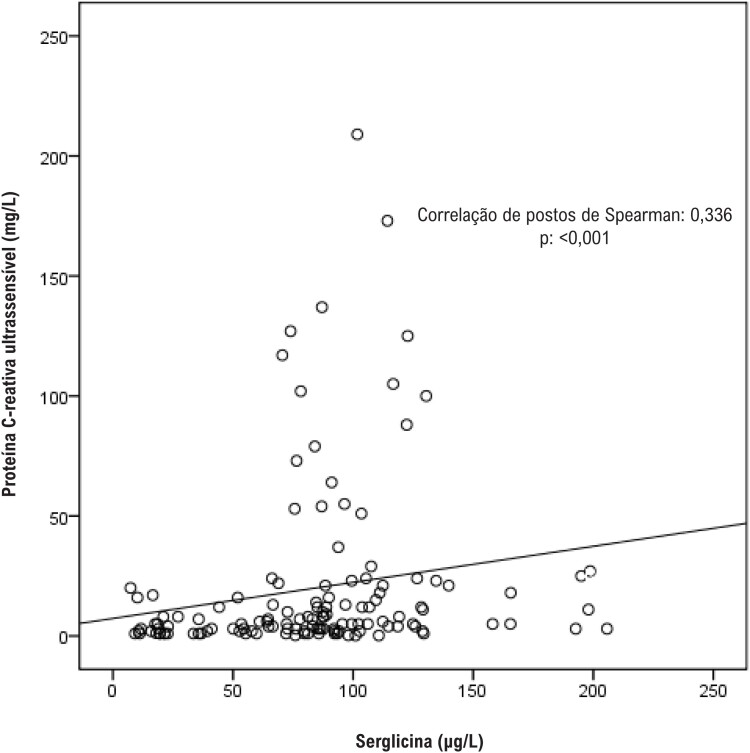
Correlações entre o nível de serglicina plasmática e o nível de proteína C-reativa ultrassensível em pacientes com IAMCSST. Houve uma correlação significativamente positiva entre o nível de serglicina plasmática e o nível de proteína C-reativa ultrassensível em pacientes com IAMCSST (r=0,336, p<0,001).

**Figura 3:**
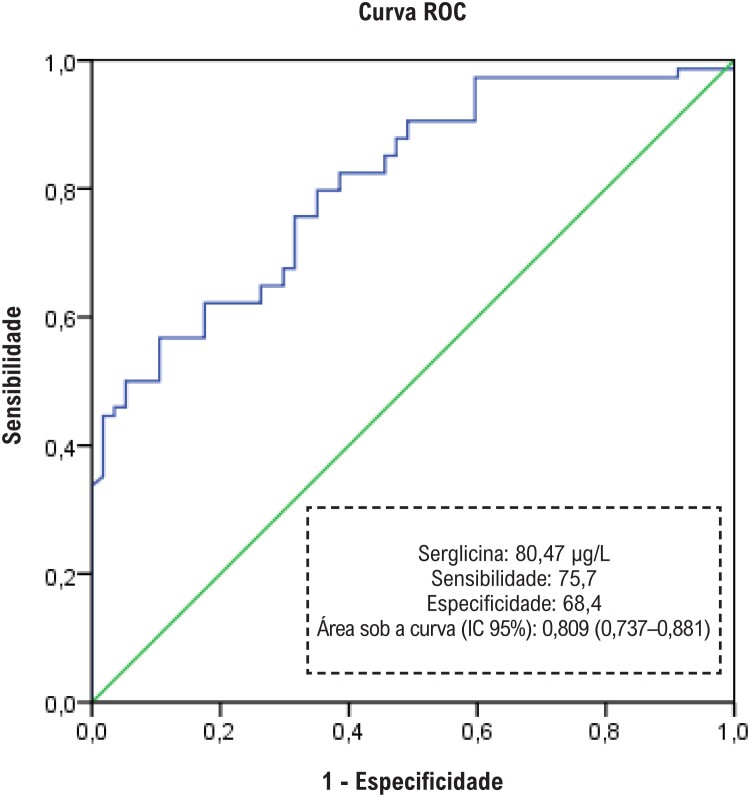
Análise ROC.

**Tabela 1:** Características basais e parâmetros laboratoriais da população estudada

	GRUPO CONTROLE (n=57)	GRUPO IAMCSST (n=75)	p
Sexo masculino, n (%)	30 (52,6)	53 (71,6)	0,025
Idade (anos)	57 (51-64)	58 (52-70)	0,253
Diabetes, n (%)	13 (22,8)	25 (33,8)	0,170
Hipertensão, n (%)	20 (35,1)	30 (40,5)	0,524
Tabagismo, n (%)	16 (28,1)	40 (66,7)	<0,001
Histórico familiar	1 (1,8)	6 (10)	0,115
FEVE (%)	64,9±0,4	45,58±10,3	<0,001
Glicemia em jejum, mg/dl	103 (94-125)	123 (98-155)	<0,001
Ureia, mg/dl	31,5 (27-36)	38 (28-49)	0,095
Creatinina, mg/dl	0,83 (0,69-0,98)	1,05 (0,9-1,17)	0,001
Hemoglobina, g/dl	14,2±2,3	13,2±2,1	0,013
Contagem de glóbulos brancos, 10/L	8 (6,8–9,6)	9,5 (8–12,3)	<0,001
Contagem de plaquetas, 10/L	266±63,7	247,5±80,1	0,154
Colesterol total, mg/dl	183,9±38,8	173,9±44,9	0,197
Triglicerídeos, mg/dl	143 (92–187)	127,5 (87,5–189,5)	0,221
HDL, mg/dl	45,6±13,7	43,2±12,8	0,329
LDL, mg/dl	107,2±36,5	101,3±36,3	0,371
PCR-us, mg/L	3 (1–6)	12 (5–29)	<0,001
Serglicina, μg/L	57,13±32,2	102,81±39,42	<0,001
Troponina, ng/L	-	4175 (1700–8690)	NA

Os dados são apresentados como média±desvio padrão, número e porcentagem (entre parênteses), ou mediana e intervalo interquartil 25–75, HDL: lipoproteína de alta densidade, PCR-us: proteína C-reativa ultrassensível, LDL: lipoproteína de baixa densidade, IAMCSST: Infarto do miocárdio com supradesnivelamento do segmento ST

**Tabela 2:** Análise univariada e multivariada mostrando os preditores de IAMCSST

Variável	Univariada		Multivariada	
	B (IC 95%)	p	B (IC 95%)	p
Sexo masculino	2,27 (1,10–4,70)	0,027	21,92 (2,58–185,76)	0,005
Tabagismo	5,12 (2,32–11,28)	<0,001	2,72 (0,59–12,59)	0,199
Idade	1,03 (0,98–1,05)	0,063	1,04 (0,98–1,11)	0,173
Glicemia em jejum	1,01 (1,07–1,02)	0,001	1,02 (1,01–1,03)	0,007
Ureia	1,03 (1,01–1,07)	0,024w\z	1,03 (0,98–1,08)	0,229
Creatinina	0,97 (0,89–1,05)	0,468		
Hemoglobina	0,81 (0,68–0,96)	0,016	0,80 (0,60–1,06)	0,129
Glóbulos brancos	1,00 (0,99–1,01)	0,625		
Proteína C-reativa ultrassensível	1,14 (1,07–1,22)	<0,001	1,17 (1,03–1,33)	0,012
Serglicina	1,04 (1,02–1,05)	<0,001	1,01 (1,00–1,01)	0,006

## Discussão

Neste estudo, descobrimos que os níveis séricos de serglicina encontravam-se significativamente elevados em pacientes com IAMCSST em comparação com indivíduos controle. Mostramos que os níveis de serglicina estavam positivamente correlacionados com os níveis de troponina e proteína C-reativa. Até onde sabemos, este é o primeiro estudo a avaliar os níveis séricos de serglicina em pacientes com IAMCSST e a demonstrar uma possível associação entre os níveis de serglicina e marcadores prognósticos em pacientes com IAMCSST.

Os proteoglicanos têm algumas funções importantes no leito vascular, incluindo a formação e organização da matriz extracelular (MEC), a regulação da interação célula-a-célula e célula-a-MEC. Assim, os proteoglicanos atuam na adesão, agregação, migração, regulação e acúmulo de lipoproteínas na hemóstase.^10^ A serglicina é um proteoglicano que pode ser sintetizado por células imunes e células endoteliais e interage com diversos mediadores como proteases, quimiocinas, citocinas e fatores de crescimento.^11^ Estudos pré-clínicos anteriores mostraram algumas evidências sobre o possível papel da serglicina na inflamação, aterogênese e trombose.

Demonstrou-se que a serglicina é expressa em todas as células imunes. A maturação das células imunes precursoras, a deposição e a liberação de diversas moléculas ativas intracelulares importantes precisam de serglicina.^12^ O fator de necrose tumoral, a interleucina 1 beta e lipossacarídeo são mediadores inflamatórios importantes e esses mediadores aumentam a síntese de serglicina.^13^ Demonstrou-se que as células deficientes em serglicina exibem uma redução significativa na produção de marcadores inflamatórios e na ativação do fator nuclear kappa beta, apesar da estimulação inflamatória.^14^ Isso estabelece que a serglicina participa da extensão da resposta inflamatória. As plaquetas também são uma fonte importante de serglicina. Anteriormente, demonstrou-se que a serglicina é o proteoglicano dominante dos grânulos alfa das plaquetas e a deficiência de serglicina resulta em defeitos de agregação e deterioração da resposta inflamatória derivada das plaquetas.^7^ A serglicina tem papel ativo nas funções endoteliais. A expressão e a secreção da serglicina apresentaram-se maiores nas células endoteliais ativadas do que nas células endoteliais quiescentes.^15^

Os dados derivados de estudos humanos sobre a serglicina são escassos e limitados; mas esses estudos fornecem evidências importantes sobre uma possível associação entre a serglicina e a doença cardiovascular aterosclerótica. Em um estudo recente, a serglicina foi encontrada entre as proteínas mais abundantemente expressas em adipócitos do tecido adiposo epicárdico em pacientes com DAC.^16^ Também se demonstrou que o fator de necrose tumoral-alfa (TNF-α) induz a expressão e secreção de serglicina no adipócito. Em outro estudo, a serglicina esteve associada à ectasia da artéria coronária, que é aceita como uma variante da doença aterosclerótica coronariana.^17^ Além disso, verificou-se que os níveis séricos de serglicina estavam correlacionados com o escore Syntax em pacientes com angina pectoris estável.^18^

Nossos resultados revelaram achados confirmatórios sobre a possível associação da serglicina com inflamação e infarto do miocárdio. Determinamos que os níveis séricos de serglicina eram maiores em pacientes com IAMCSST do que em indivíduos controle. Os níveis séricos de serglicina estiveram positivamente correlacionados com os níveis máximos de troponina e os níveis de proteína C-reativa ultrassensível. Não está claro a partir de nossos resultados se a elevação da serglicina é uma causa de infarto do miocárdio ou um achado secundário devido à resposta inflamatória ou infarto. Embora nossos resultados não deem uma explicação clara sobre a relação entre a patogênese do IAMCSST e a serglicina, o presente estudo fornece dados preciosos sobre a associação entre os níveis de serglicina com a inflamação e o tamanho do infarto.

### Limitações do estudo

Os achados do nosso estudo devem ser interpretados com algum cuidado devido às limitações descritas a seguir. Trata-se de um estudo transversal, de pequena escala e unicêntrico. Não coletamos dados sobre desfechos concretos como morte ou insuficiência cardíaca sintomática, portanto, não podemos comentar a associação entre os níveis de serglicina e eventos cardiovasculares adversos em pacientes com IAMCSST. Além disso, não coletamos dados que reflitam o prognóstico de IAMCSST como escore TIMI, escore GRACE, classe Killip ou níveis de peptídeo natriurético do tipo-B (BNP). Mas acreditamos que este estudo forneça informações significativas, demonstrando a associação da serglicina com a proteína C-reativa ultrassensível e os níveis máximos de troponina. Não fizemos medições seriadas de serglicina sérica em pacientes com IAMCSST. Sendo assim, neste estudo, é impossível tecer quaisquer comentários sobre como os níveis de serglicina mudam no decorrer do infarto do miocárdio.

## Conclusões

O presente estudo apresenta dois achados principais. Um deles é a associação entre a serglicina e a resposta inflamatória demonstrada pela proteína C-reativa ultrassensível. O outro é a associação entre serglicina e o tamanho do infarto, demonstrada pelos níveis máximos de troponina. Nossos resultados podem ser uma fonte de inspiração para estudos que avaliem o papel da serglicina na patogênese da síndrome coronariana aguda. Somos da opinião de que estudos adicionais e mais exaustivos são necessários para esclarecer melhor a relação entre a serglicina e IAMCSST.

## References

[1] 1. Zakynthinos E, Pappa N. Inflammatory biomarkers in coronary artery disease. J Cardiol. 2009;53(3):317–33. 10.1016/j.jjcc.2008.12.007 19477372

[2] 2. Hansson GK. Inflammation, Atherosclerosis, and Coronary Artery Disease. N Engl J Med [Internet]. 2005;352(16):1685–95. Available from: https://www.nejm.org/doi/full/10.1056/NEJMra043430 10.1056/NEJMra043430 15843671

[3] 3. Kolset SO, Tveit H. Serglycin - Structure and biology. Cell Mol Life Sci. 2008;65(7–8):1073–85. 10.1007/s00018-007-7455-6 PMC11131666 18066495

[4] 4. Zernichow L, Åbrink M, Hallgren J, Grujic M, Pejler G, Kolset SO. Serglycin is the major secreted proteoglycan in macrophages and has a role in the regulation of macrophage tumor necrosis factor-α secretion in response to lipopolysaccharide. J Biol Chem. 2006;281(37):26792–801. 10.1074/jbc.M512889200 16807245

[5] 5. Reine TM, Vuong TT, Jenssen TG, Kolset SO. Serglycin secretion is part of the inflammatory response in activated primary human endothelial cells in vitro. Biochim Biophys Acta - Gen Subj [Internet]. 2014;1840(8):2498–505. Available from: http://dx.doi.org/10.1016/j.bbagen.2014.02.002 10.1016/j.bbagen.2014.02.002 24513305

[6] 6. Kolseth IBM, Reine TM, Vuong TT, Meen AJ, Fan Q, Jenssen TG, et al. Serglycin is part of the secretory repertoire of LPS-activated monocytes. Immunity, Inflamm Dis. 2015; 10.1002/iid3.47 PMC4386912 25866637

[7] 7. Woulfe DS, Lilliendahl JK, August S, Rauova L, Kowalska MA, Åbrink M, et al. Serglycin proteoglycan deletion induces defects in platelet aggregation and thrombus formation in mice. Blood. 2008;111(7):3458–67. 10.1182/blood-2007-07-104703 PMC2275015 18094327

[8] 8. Schick BP. Serglycin Proteoglycan Deletion in Mouse Platelets : Physiological Effects and Their Implications for Platelet Contributions to Thrombosis, Inflammation, Atherosclerosis, and Metastasis I. Progr Mol Biol Trans Sci.2010;93:235-7. 10.1016/S1877-1173(10)93011-1 20807648

[9] 9. Thygesen K, Alpert JS, Jaffe AS, Simoons ML, Alpert JS, White HD, et al. Third universal definition of myocardial infarction. Eur Heart J. 33(20): 2012;2551–67. 10.1093/eurheartj/ehs184 22922414

[10] 10. Chang MY, Chan CK, Braun KR, Green PS, Brien KDO, Chait A, et al. Monocyte-to-Macrophage Differentiation SYNTHESIS AND SECRETION OF A COMPLEX EXTRACELLULAR MATRIX *. 2012;287(17):14122–35. 10.1074/jbc.M111.324988 PMC3340194 22351750

[11] 11. Kolset SO, Pejler G. Serglycin: A Structural and Functional Chameleon with Wide Impact on Immune Cells. J Immunol [Internet]. 2011;187(10):4927–33. Available from: http://www.jimmunol.org/lookup/doi/10.4049/jimmunol.1100806 10.4049/jimmunol.1100806 22049227

[12] 12. Scully OJ, Chua P, Harve KS, Bay B, Yip GW. Serglycin in Health and Diseases. Anat Rec{Hoboken).2012;295(9):1415-20. 10.1002/ar.22536 22807344

[13] 13. Korpetinou A, Skandalis SS, Labropoulou VT, Smirlaki G, Noulas A, Karamanos NK, et al. Serglycin: At the Crossroad of Inflammation and Malignancy. Front Oncol [Internet]. 2014;3(January):1–12. Available from: http://journal.frontiersin.org/article/10.3389/fonc.2013.00327/abstract 10.3389/fonc.2013.00327 PMC3888995 24455486

[14] 14. Scuruchi M, Ascola AD, Avenoso A, G GM, S SC, Campo GM. Contributed equally to this work. Arch Biochem Biophys. 2019;

[15] 15. Reine TM, Vuong TT, Rutkovskiy A, Meen AJ, Vaage J. Serglycin in Quiescent and Proliferating Primary Endothelial Cells. 2015;1–28. 10.1371/journal.pone.0145584 PMC4687888 26694746

[16] 16. Imoto-Tsubakimoto H, Takahashi T, Ueyama T, Ogata T, Adachi A, Nakanishi N, et al. Serglycin is a novel adipocytokine highly expressed in epicardial adipose tissue. Biochem Biophys Res Commun [Internet]. 2013;432(1):105–10. Available from: http://dx.doi.org/10.1016/j.bbrc.2013.01.078 10.1016/j.bbrc.2013.01.078 23376071

[17] 17. Kundi H, Gök M, Topçuoglu C, Ornek E. Związek stężenia serglicyny z izolowanym tętniakowatym poszerzeniem tętnic wieńcowych. Kardiol Pol [Internet]. 2017;75(10):990–6. Available from: https://ojs.kardiologiapolska.pl/kp/article/view/11101 10.5603/KP.a2017.0119 28612914

[18] 18. Bolayir HA, Kivrak T, Gunes H, Bolayir A, Karaca I. The association between serum serglycin level and coronary artery disease severity in patients with stable angina pectoris. Kardiol Pol. 2018;76(4):783-96. 10.5603/KP.2018.0007 29313562

