# Supplementary material for: Associação entre os Níveis Séricos se Serglicina e o Infarto do Miocárdio com Supradesnivelamento do Segmento ST
Source: Arq Bras Cardiol. 2021 Apr 8;116(4):756–62. [Article in Portuguese] doi: 10.36660/abc.20190554 (PMC8121402; doi:10.36660/abc.20190554)
Supplement: Translation

# Translation: Association between Serum Serglycin Levels and St-Segment Elevation Myocardial Infarction

**Authors:** Burcu Ugurlu Ilgın, Emrullah Kızıltunç, Murat Gök, Ender Ornek, Canan Topcuoglu, Mustafa Çetin, Orhan Karayiğit

**Affiliations:** 1 Ankara Turkey TC Saglık Bakanlıgı Gazi Mustafa Kemal Devlet Hastanesi – Cardiology, Ankara - Turkey; 2 Edirne Turkey Cardiology Department, Edirne Provincial Health Directorate Edirne Sultan 1st Murat State Hospital, Edirne - Turkey; 3 Ankara Turkey Medical Biochemistry Department, Numune Education and Research Hospital, Ankara - Turkey; 4 Ankara Turkey Cardiology Department, Numune Education and Research Hospital, Ankara - Turkey

**Keywords:** Cardiovascular Diseases, Myocardial Infarction, Atherosclerosis, Coronary Artery Disease, Inflammation, Biomarkers, Serglycin

## Abstract

**Background:**

It is suggested that serglycin has important functions in fibrin stabilization and inflammation but there is limited information on its clinical value for atherosclerotic heart disease.

**Objective:**

The purpose of this study is to find out serum serglycin levels in acute myocardial infarction patients and in the control group individuals; and to investigate the association between serglycin levels with inflammation markers and infarct size markers.

**Methods:**

The study population consisted of 75 patients with ST-segment elevation myocardial infarction (STEMI) and 57 patients with normal coronary arteries (NCA) (control group). Patient characteristics, serum serglycin levels, high-sensitivity C-reactive protein (hs-CRP) levels, peak troponin T levels and other biochemical parameters were recorded. A p value <0.05 was considered statistically significant.

**Results:**

The control group consisted of individuals who are younger and smoke less than those of the STEMI group. The number of females in the control group was higher than in the STEMI group. Serum serglycin levels were significantly higher in the STEMI group than in control group (102.81±39.42 vs. 57.13±32.25, p<0.001). Correlation analyses revealed a significant positive correlation between serglycin and troponin (Spearman's Rho: 0.419; p<0.001) and between serglycin and hs CRP (Spearman's Rho: 0.336; p<0.001). Multivariate logistic regression analysis demonstrated that serum serglycin levels were independently associated with STEMI. Using a cutoff level of 80,47 μg/L, the serglycin level predicted the presence of STEMI with a sensitivity of 75.7% and specificity of 68.4%.

**Conclusion:**

Serum serglycin levels were significantly higher in the STEMI group than in the control group. Serum serglycin levels were positively correlated with both hs CRP levels and troponin levels.

## Introduction

Atherosclerotic heart disease is one of the most important causes of death and morbidity all around the world. Chronic vascular inflammation is accepted atherosclerotic plaque formation, but the promoters and drivers of chronic vascular inflammation are still under investigation.^1^^,^^2^

Serglycin is an intracellular proteoglycan expressed mostly in neutrophils, lymphocytes, monocytes, macrophages, platelets, megakaryocytes and mast cells,^3^ but it can also be produced by certain non-hematopoietic cells like endothelial cells.^4^ It is stored in cell vesicles and reacts with mediators such as cytokines, chemokines, growth factors and proteases.^3^ There is evidence about the role of serglycin in inflammation and atherogenic-prothrombotic cascades. It was demonstrated that serglycin synthesis and secretion are triggered in human endothelial cells and monocytes by proinflammatory stimulants.^5^^,^^6^ In another study, it was found that serglycin binds to C1q receptors, and affects fibrin polymerization in fibrin clot formation.^7^ Serglycin is one of the ingredients of platelet alfa granules. These granules are involved in platelet activation in response to inflammation, thrombus formation and atherosclerosis.^8^

The aforementioned effects and functions of serglycin lead to high suspicions about the potential relationship between serglycin and atherosclerotic cardiovascular disease, but there is not good evidence. Therefore, this study aimed to investigate serum serglycin levels in ST-segment elevation myocardial infarction (STEMI) patients and to evaluate an association between serum serglycin levels and prognostic markers of STEMI.

## Methods

### Study population

We enrolled patients with acute ST-segment elevation myocardial infarction (STEMI) and patients with normal coronary arteries in this single center cross-sectional study between November 2017 and March 2018 at Numune Education and Research Hospital, Ankara, Turkey. The study protocol was approved by the local ethical committee and informed consent forms were obtained from all participants.

STEMI diagnosis were made according to the third universal definition of myocardial infarction document.^9^ All STEMI patients underwent primary percutaneous coronary intervention and received guided medical treatment according to contemporary scientific knowledge. Patients who underwent elective coronary angiography and were found to have normal coronary arteries were included in the study as the control group. All STEMI patients and patients with normal coronary arteries were consecutively recruited in the study. Acute coronary syndrome patients without a STEMI diagnosis were excluded from the study. Patients with any hematological disorder, chronic inflammatory disease, previous stroke, stable coronary artery disease, heart failure, renal disease, liver disease, malignancy, rheumatological disease, previous myocardial infarction or history of coronary artery surgery were also excluded. Transthoracic echocardiography was performed on all participants. Left ventricular ejection fraction was calculated using the Simpson's method.

### Laboratory tests

All blood samples for serglycin analysis were collected from the patients after angiography, into plain tubes, and the serum was separated by centrifugation at 4000 rpm for 10 min and stored at −80 °C. Complete differential blood counts were determined in peripheral venous blood samples obtained upon admission. An automated analyzer was used to measure troponin, high-sensitivity C-reactive protein (hs-CRP), total cholesterol, triglyceride, creatinine and low- and high-density lipoprotein cholesterol levels. Serum serglycin levels were measured by a human serglycin enzyme-linked immunosorbent assay kit (Lot No.: E17-109S01, BioVendor Research and Diagnostic Products, 62100 Bmo, Czech Republic). All samples were processed simultaneously.^5^ The coefficients of variation (CV) of the kit were 3.7% and 2.9% for 57.77 ng/mL and 81.57 ng/mL concentrations, respectively, and the sensitivity was 9.5 ng/mL.

### Statistical analysis

The software package SPSS 22.0 was used to perform all statistical analyses. Distribution of the variables was analyzed using the Kolmogorov–Smirnov test. Continuous data were presented as means ± standard deviation or as medians with interquartile ranges, depending on the distribution pattern. Independent-samples t-test was used to compare parametric continuous variables and the Mann–Whitney U-test was used to compare nonparametric continuous variables. Categorical variables were compared using the chi-square test and expressed as percentages. The correlation between hs-CRP and serglycin levels was assessed by Spearman's rank test. For the multivariate analysis, the possible factors identified in univariate analysis were further entered into the logistic regression analysis to determine the independent predictors of myocardial infarction. The capacity of serum serglycin levels in predicting STEMI were analyzed using the ROC (receiver operating characteristic) curve analysis. While evaluating the area under curve, a 5% type-I error level was used to accept a statistically significant predictive value of the test variable. As there was no data about serglycin levels in coronary artery disease patients in the literature written in English, we were not able to calculate sample size before the study.

## Results

A total of 132 patients (75 STEMI and 57 NCA) were included in the study. The clinical characteristics and biochemical parameters of the STEMI and control groups are presented in Table 1. Male patient ratio and smoking rate were higher in the STEMI group. Patients were younger in the control group than in the STEMI group. Serum serglycin levels were significantly higher in the STEMI group than in the control group (Table 1). Serum serglycin levels were significantly correlated with troponin (r=0.419, p<0.001) and hs-CRP (r=0.336, p<0.001; Figure 1 and 2) levels. Logistic regression analyses revealed that gender (male), fasting blood glucose level, hs CRP and serglycin levels were independent predictors of STEMI (Table 2). ROC analysis was performed to determine the serglycin level capability to predict STEMI. The area under the curve was 0.809 (95% confidence interval: 0.737–0.881; p<0.001). Using a cutoff level of 80.47 *μ*g/L, the serglycin level predicted the presence of STEMI with a sensitivity of 75.7% and specificity of 68.4% (Figure 3).

**Figure 1:** Correlations between plasma serglycin level and troponine level in STEMI patients. There was a significantly positive correlation between plasma serglycin level and troponine level in STEMI patients (r=0.419, p<0.001).

**Figure 2:** Correlations between plasma serglycin level and hs-CRP level in STEMI patients. There was a significantly positive correlation between plasma serglycin level and hs-CRP level in STEMI patients (r=0.336, p<0.001).

**Figure 3:** ROC analysis.

**Table 1:** Baseline characteristics and laboratory parameters of the study population

	CONTROL GROUP (*n*=57)	STEMI GROUP (*n*=75)	p
Male, *n (%)*	30 (52.6)	53 (71.6)	0.025
Age (years)	57 (51-64)	58 (52-70)	0.253
Diabetes, *n (%)*	13 (22.8)	25 (33.8)	0.170
Hypertension, *n (%)*	20 (35.1)	30 (40.5)	0.524
Smoking, *n (%)*	16 (28.1)	40 (66.7)	<0.001
Family history	1 (1.8)	6 (10)	0.115
LVEF (%)	64.9±0.4	45.58±10.3	<0.001
Fasting blood glucose, mg/dl	103 (94-125)	123 (98-155)	<0,001
Urea, mg/dl	31.5 (27-36)	38 (28-49)	0.095
Creatinine, mg/dl	0.83 (0.69-0.98)	1.05 (0.9-1.17)	0,001
Hemoglobin, g/dl	14.2±2.3	13.2±2.1	0.013
White blood cell count, 10/L	8 (6.8–9.6)	9.5 (8–12.3)	<0.001
Platelet count, 10/L	266±63.7	247.5±80.1	0.154
Total cholesterol, mg/dl	183.9±38.8	173.9±44.9	0.197
Triglycerides, mg/dl	143 (92–187)	127.5 (87.5–189.5)	0.221
HDL, mg/dl	45.6±13.7	43.2±12.8	0.329
LDL, mg/dl	107.2±36.5	101.3±36.3	0.371
hs-CRP, mg/L	3 (1–6)	12 (5–29)	<0.001
Serglycin, μg/L	57.13±32.2	102.81±39.42	<0.001
Troponin, ng/L	-	4175 (1700–8690)	NA

Data are presented as mean ± standard deviation, number and percentage (in brackets), or median and interquartile range 25–75. HDL: high density lipoprotein, hs-CRP: high-sensitivity C-reactive protein, LDL: low density lipoprotein, STEMI: ST-segment elevation myocardial infarction

**Table 2:** Univariate and multivariate analysis showing the predictors of STEMI

Variable	Univariate		Multivariate	
	B (95% CI)	p	B (95% CI)	p
Male gender	2.27 (1.10–4.70)	0.027	21.92 (2.58–185.76)	0.005
Smoking	5,12 (2.32–11.28)	<0.001	2.72 (0.59–12.59)	0.199
Age	1.03 (0.98–1.05)	0.063	1.04 (0.98–1.11)	0.173
Fasting blood Glucose	1.01 (1.07–1.02)	0.001	1.02 (1,01–1,03)	0.007
Urea	1.03 (1.01–1.07)	0.024	1.03 (0.98–1.08)	0.229
Creatinine	0.97 (0.89–1.05)	0.468		
Hemoglobin	0.81 (0.68–0.96)	0.016	0.80 (0.60–1.06)	0.129
White blood cell	1.00 (0.99–1.01)	0.625		
hs CRP	1.14 (1.07–1.22)	<0.001	1.17 (1.03–1.33)	0.012
Serglycin	1.04 (1.02–1.05)	<0.001	1,01 (1,00–1,01)	0.006

## Discussion

In this study, we found that serum serglycin levels were significantly increased in STEMI patients compared to control individuals. We showed that serglycin levels were positively correlated with troponin levels and CRP levels. To the best of our knowledge, this is the first study evaluating serum serglycin levels in STEMI patients and demonstrating a potential association between serglycin levels and prognostic markers in patients with STEMI.

Proteoglycans have some important functions in vascular bed, including extracellular matrix (ECM) formation and organization, regulation of cell-to-cell and cell-to-ECM interaction. Thus, proteoglycans functions in hemostasis adhesion, aggregation, migration, regulation and lipoprotein accumulation.^10^ Serglycin is a proteoglycan which can be synthesized by immune cells and endothelial cells and it interacts with numerous mediators such as proteases, chemokines, cytokines and growth factors.^11^ Previous preclinical studies showed some evidence about the potential role of serglycin in inflammation, atherogenesis and thrombosis.

It was demonstrated that serglycin is expressed in all immune cells. Maturation of precursor immune cells, deposition and release of many important intracellular active molecules need serglycin.^12^ Tumor necrosis factor, interleukin 1 beta and liposaccharide are important inflammatory mediators and these mediators upregulate serglycin synthesis.^13^ It was demonstrated that serglycin deficient cells exhibit significantly decreased inflammatory marker production and nuclear factor kappa beta activation despite inflammatory stimulation.^14^ This establishes that serglycin participates in the extension of inflammatory response. Platelets are also an important source of serglycin. It was previously demonstrated that serglycin is the dominant proteoglycan of the platelet alfa granules and serglycin deficiency results in aggregation defects and deteriorated platelet-derived inflammatory response.^7^ Serglycin has an active role in endothelial functions. Serglycin expression and secretion was found to be higher in activated endothelial cells than in quiescent endothelial cells.^15^

Data derived from human studies about serglycin are meagre and limited; but these studies provide important evidence about a potential association between serglycin and atherosclerotic cardiovascular disease. In a recent study, serglycin was found to be among the most abundantly expressed proteins in adipocytes of epicardial adipose tissue in patients with CAD.^16^ It was also demonstrated that the tumor necrosis factor-α (TNF-α) induces the expression and secretion of serglycin in adipocyte. In another study, serglycin was found to be associated with coronary artery ectasia, which is accepted as a variant of coronary atherosclerotic disease.^17^ In addition, it was found that serum serglycin levels were correlated with Syntax score in patients with stable angina pectoris.^18^

Our results revealed confirmatory findings about the potential association of serglycin with inflammation and myocardial infarction. We determined that serum serglycin levels were higher in STEMI patients than in control individuals. Serum serglycin levels were positively correlated with peak troponin levels and hs CRP levels. It is not clear from our results whether serglycin elevation is a cause of myocardial infarction or it is a secondary finding due to inflammatory response or infarction. Although our results fail to give a clear explanation of the relationship between STEMI pathogenesis and serglycin, this study provides precious data about the association between serglycin levels with inflammation and infarction size.

### Limitations of the study

The findings of our study should be interpreted with some caution due to the following limitations. This was a single-center, small-scale, cross sectional study. We did not collect any data about hard outcomes like death or symptomatic heart failure so we cannot make any comments about the association between serglycin levels and adverse cardiovascular events in STEMI patients. Besides, we did not gather any data reflecting the prognosis of STEMI like TIMI score, GRACE score, Killip class or BNP levels. But we believe that this study provides significant information by demonstrating the association of serglycin with hs CRP and peak troponin levels. We did not make serial serum serglycin measurements in STEMI patients. So, it is impossible to make any comments about how serglycin levels change in the course ofmyocardial infarction with this study.

## Conclusions

This study has two major findings. One is the association between serglycin and inflammatory response demonstrated by hs CRP. The other is the association between serglycin and infarct size demonstrated by peak troponin levels. Our results may be a source of inspiration for studies evaluating the role of serglycin in acute coronary syndrome pathogenesis. We are of the opinion that further, more extensive studies are needed to further clarify the relationship between serglycin and STEMI.
